# Vena cava thrombosis associated with nephrotic syndrome in the puerperal gestational cycle

**DOI:** 10.1590/S1516-31802001000100007

**Published:** 2001-01-02

**Authors:** Maíta Poli Araújo, Carla Gonçalves, Roberta Gonçalves, José Wilson Ramos Braga, Thaís Vilela Peterson, Álvaro Nagib Atallah, Emília Inoue Sato, Virgínia Fernandes Moça Trevisani

**Keywords:** Nephrotic Syndrome, Puerperium, Pregnancy, Cava thrombosis, Thromboembolism, Síndrome nefrótica, Puerpério, Gravidez, Trombose da veia cava, Tromboembolismo

## Abstract

**CONTEXT:**

The puerperal gestational cycle is accompanied by a state of physiological hypercoagulability. Thromboembolic phenomena may occur at this time.

**OBJECTIVE:**

To report on a clinic case involving a patient that presented a family history of thromboembolism and developed deep vein thrombosis in a lower limb and vena cava thrombosis during the puerperal gestational cycle, displaying nephrotic syndrome as the main complication.

**DESIGN:**

Case report.

## RESUMO

**CONTEXTO:**

A gestação é acompanhada de um estado fisiológico de hipercoagulabilidade. Fenômenos de Trombose podem ocorrer.

**OBJETIVO:**

Relatar um caso clínico envolvendo uma paciente que apresentava história importante de trombifilia familiar, e que desenvolveu trombose venosa profunda no membro inferior e trombose da veia cava durante o ciclo gravídico-puerperal, apresentando síndrome nefrótica como principal complicação.

**TIPO DE ESTUDO:**

Relato de caso.

## INTRODUCTION

There are some situations that predispose towards a state of hypercoagulability. In nephrotic syndrome, in the puerperal gestational cycle and in states of thrombophilia, alterations occur in coagulation factors and in the physiological inhibitions of coagulation that may lead to the formation of thrombus.^1-4^ A thrombus situated in the inferior vena cava, at the junction of the renal veins, may compromise renal function through the pressure increase. Although deep vein thrombosis is easy to recognize, the etiologic diagnosis is still complicated, mainly due to the existence of many predisposal factors. Thus, knowledge of deep vein thrombosis physiopathology and the risk factors related to the clinic condition are important, so that a standard diagnostic study and suitable therapy may be introduced as soon as possible, avoiding complications such as pulmonary embolism.

This work has the aim of reporting on a clinic case involving a patient that presented a family history of thromboembolism and developed deep vein thrombosis in a lower limb and vena cava thrombosis during the puerperal gestational cycle, displaying nephrotic syndrome as the main complication.

## CASE REPORT

The patient, a white 19-year-old married housewife from São Paulo, Brazil, entered the hospital complaining of pains and swelling in the right leg. She had suffered these pains for three days. Her clinical history showed that she had had a baby via vaginal delivery in hospital, without complications, one month and half earlier. She was primipara and this was her first gestation. During the gestation, she did prenatal screening, and apparently there were no abnormalities.

In the immediate puerperium, she presented urinary infection that was treated using norfloxacin. The pain that led her to the hospital started in the right knee and thereafter followed a descending course to the foot, followed by the development of accentuated edema. She also felt paresthesia, burning, and the sensation of fever, anorexia, and asthenia.

In relation to the various systems, she reported frontal headache, rotatory vertigo when sudden movements were made, dry cough, pain in the knees, and alteration in the color of the hands, that sometimes were pale, reddish or purplish.

She denied having antecedents of hepatitis, diabetes, tuberculosis, hypertension, sexually transmitted diseases, drug taking, and surgeries or previous internment. As far as the family history was concerned, she mentioned that her father had hypertension and diabetes, and he had had deep vein thrombosis in the right lower limb when he was 38. He was treated using anticoagulant and elastic socks. He died of a cerebral vascular accident when he was 55. The mother (47 years old) was alive and healthy. The patient had three healthy brothers, and four sisters. The eldest one, 32, had had three miscarriages and during her last gestation she presented thrombosis in a lower limb and had to be submitted to anticoagulant therapy for six months. She denied having antecedents of tumors, malformations, or connective tissue disorders.

**Figure 1:**
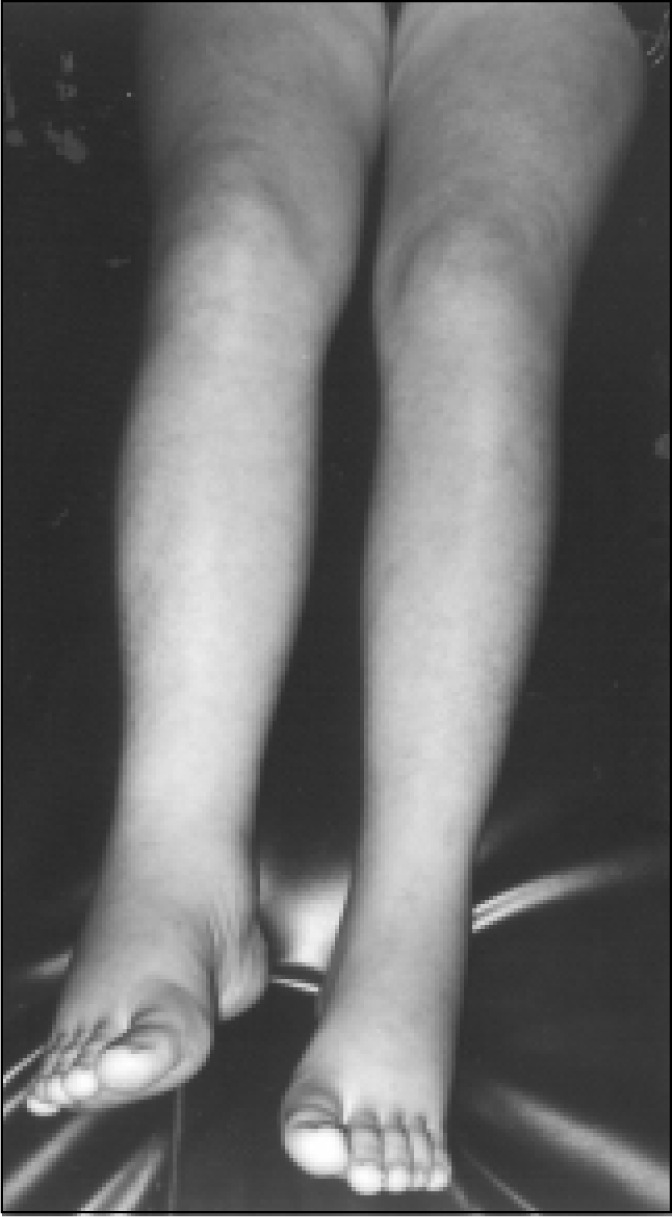
Lower limbs. Observe reticular livedo and right lower limb edema.

**Figure 2:**
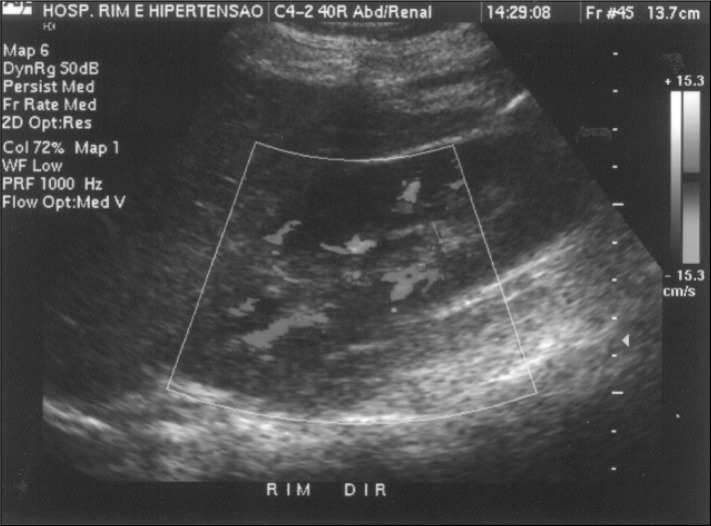
Right renal Doppler ultrasound showing inferior cava thrombosis including the area of the junction of the renal veins.

**Figure 3:**
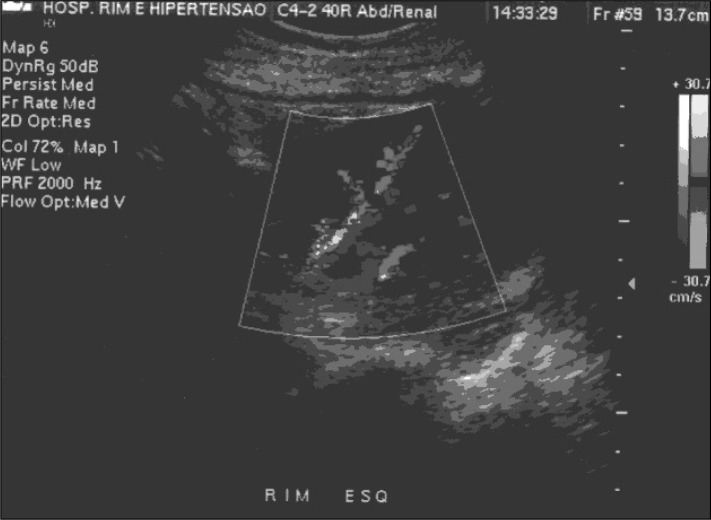
Left renal Doppler ultrasound : inferior cava thrombosis.

During the examination in the hospital, the patient showed a regular general state, cutaneous paleness, colorless mucous, and was hydrous, acyanotic, without icterus, without fever, eupneic, orientated and contacting normally. Blood pressure was 110x70 mmHg; cardiac frequency 120 bpm; respiratory rate 15 rpm. In her face, a trace of gestational chloasma could be seen, with lymph nodes not evident, thyroid not evident and absence of jugular stasis (Figure 1). She did not present cardiorespiratory or abdominal alterations. In the superior extremities she did not present any abnormality. In the inferior extremities, reticular livedo could be seen in both limbs, with edema and significant pain in the right lower limb from the base of the thigh, with pasting and difficulty in dorsiflexion of the foot (Homans sign). The left lower limb was normal (Figures 2 and 3). Palpation of the peripheral pulses (popliteus, pedious) showed that the left side was normal and the right side, slightly decreased.

The patient was interned with the following diagnostic theories: puerperal hypercoagulability, antiphospholipid antibody syndrome, nephrotic syndrome, puerperal anemia, infection of the uri-nary system and connective tissue disorder. At the time of internment, laboratory tests showed the following: urine I: 8,800 leukocytes/ml; hematuria: 16,000/ml, with erythrocytic dimorphism, some hyaline casts, some bacteria, many epithelial cells, positive mucus filaments, negative glucose and proteinuria (8.2 g/l). Blood count: 15,800 leukocytes/mm^3^; with the differentiation being 1% band forms, 94% segmented, 1% eosinophils, 0% basophils, 3% lymphocytes and 1% monocytes; microcytic hypochromic anemia with hemoglobin of 7.8 g/dl; hematocrit of 27% and platelets: 170,000 mm^3^.

The coagulation tests presented prothrombin time (PT) = 74%; bleeding time (BT) = 3 minutes; activated partial thromboplastin time (APTT) = 32.9 s; ratio APTT patient/APTT pool = 1.18.

The biochemistry of the blood presented: creatine = 1.1 mg/dl; potassium = 4.8 Eq/l; sodium = 135 mEq/l; total bilirubin = 0.4 mg/dl (direct = 0.2 md/dl); glucose = 139 mg/dl.

The venous gasometry presented: pH = 7.37; pCO_2_ = 36 mmHg; pO_2_ = 35 mmHg; HCO_3_ = 23 mmol/L; BE = 3.

Proteinuria (24 hours) = 3.23 g in a volume of 320 ml (10.1 g/L); creatinine depuration = 51 ml/min; ALT = 17u/L; AST = 21 u/L; CK = 56 u/L; CKMB =20 u/L. Direct and indirect Coombs were negative. The dosage of antithrombin III was dosed using the functional method with chromogen substrate S2765, with a result of 135% of activity (normal range: 80 to 120%).

From the clinical and laboratory data, the following could be diagnosed: deep vein thrombosis, urinary infection, nephrotic syndrome, and probable systemic lupus erythematosus. Anticoagulant intravenous therapy with heparin was introduced for 6 days, with warfarin sodium (Marevan^R^) from the third day. Anticoagulant therapy control was performed using a daily dosage of APTT and, even with daily dosages of 1,600 u/h of heparin, the APTT did not alter.

**Figure 4:**
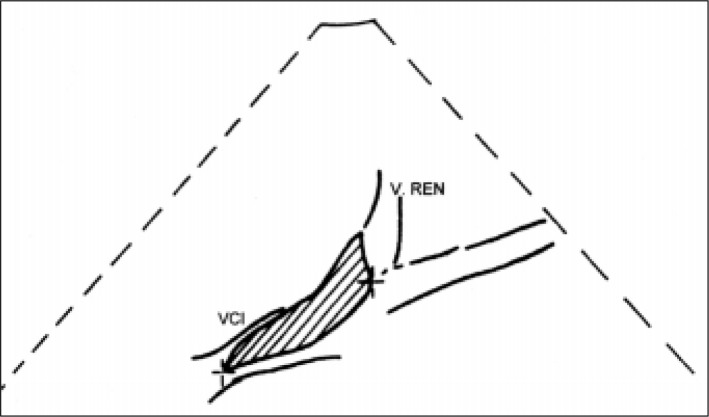
Showing the probable location of the thrombosis. V. Ren: Renal vein; VCI: Inferior cava vein.

Treatment using prednisone (Meticorten^R^ 60 mg/day) was also introduced. On the tenth day, renal Doppler ultra-sound was requested, and inferior vena cava thrombosis was found, including the area of the junction of the renal veins. It was not possible to identify flux in this topography. The kidneys were morphologically preserved, with normal dimensions, diffuse decrease of their echo texture and loss in parenchyma-sinus differentiation. Neither hydro-nephrosis nor calculus was identified. The right kidney measured 11.6 x 4.1 cm at its largest diameters and the left kidney measured 10.4 x 4.9 cm (Figure 4).

Antinuclear factor, anti-ENA (extractable nuclear antigens), anti-DNA native, anti-cardiolipin, lupus anticoagulant and cryoglobulin were negative.

On the fourteenth day, serum albumin (1.58 g/dL) was observed and therapy of three flasks of albumin EV was introduced. The 24-hour proteinuria presented a decrease (from 10.1 to 1.1 g/L – 1.36g in a volume of 1240 ml corresponding to 24 hours) and the creatinine depu-ration increased (from 51 ml/min to 69 ml/min). The urine test I that had presented proteinuria of 8.2 g/L went to 0.9 g/L and the erythrocytes that were 100/field decreased to 10/field.

The patient was interned for 28 days with the following evolution. On the second day of internment there was an intensification of the articulation pains in the right lower limb and she also started to present discrete edema in the left lower limb. Appearance of malar rash, bilateral bipalpebral edema and fever were also observed. The pain had progressive improvement with medications, but the generalized edema was kept till the fourteenth day, when it started to regress slowly. By the time she was discharged, on the twenty-eighth day, there was no more pain or edema.

The nephrotic syndrome progressed with improvement on the fourteenth day, when remission of the thromboembolic condition started. The patient left the hospital with no symptoms. On the day she was discharged, a new ultrasound of the renal veins and vena cava was done, revealing the presence of normal-caliber vena cava and renal veins, showing no signs of thrombosis. Both the left and right kidneys presented normal configuration. The anticoagulant medication was maintained and planned to continue for 6 months, using warfarin sodium (Marevan^R^ 2 tablets a day) and prednisone (Meticorten^R^ 10 mg a day).

The patient was clinically followed up with periodic checks of PT and INR (international normative relation) between 2.0 and 3.0 (approximately 30% of the prothrombin activity), and orientated in relation to the risks for new thromboembolic phenomena.

A month after she left the hospital, the patient presented a new condition in the deep vein system, now in the left lower limb. New treatment with anticoagulant was introduced and clinical tests were requested, which presented the following values:

Protein C activity = 77% (normal range: 70 to 130%); free protein S in the plasma: 25% (normal = 70 to 130%); APTT =45.9 seconds (APTT pool normal plasma = 29.2 seconds); relation APTT patient/APTT pool = 1.57; dRVVT (dilute Russell's Viper Venom test) = 46.2 seconds; normal plasma = 30.5 seconds; relation dRVVT patient/dRVVT pool normal plasma = 1.31 (lupus anticoagulant; relation of dRVVT > 1.14), PNP test (platelet neutralization process); PNP patient = 43.2 seconds; PNP control = 51.8 seconds; reference value = PNP extension control > PNP patient = lupus anticoagulant; ATIII = 92% (normal = 80 to 120%); resistance test to activated Protein C in addition to plasma deficient of factor V: result = 52.3, indicating absence of resistance to protein C; Leiden factor V: absent; homocysteine = 36.5 mol/l (normal = 5.5 to 17.0 mol/L).

Analyzing the clinical and laboratory data, a diagnosis of antiphospholipid antibody syndrome and hyperhomocysteinemia was made, and the patient was treated with anticoagulant therapy and frequent clinical attendance.

## DISCUSSION

Reviewing the risk factors for venous thromboembolic disease, it can be seen that this is about five times more frequent during pregnancy, and is one important reason for obstetric morbidity and mortality.^1^ The risk is present throughout pregnancy, but it becomes increased especially in the last trimester as well as in the five weeks after childbirth. The main reason for this is the venous stasis associated with the increase in the level of coagulation factors. On the other hand, the factors that block coagulation are decreased.^1-5^ As this patient was in the late puerperium, and might have been in a state of hypercoagulability, the occurrence of deep vein thrombosis can be explained.

The nephrotic syndrome may lead to hypercoagulability as a consequence of the alteration in the level of coagulation factors, including a decrease in factors IX, X, and XII; elevation especially of the levels of the factors V and VIII, fibrinogen, beta-thromboglobulin and platelets; decrease in coagulation inhibitors, such as proteins C and S and ATIII; and increase in platelet aggregation.

Leiden factor V may be associated with complications during pregnancy, such as thromboembolism, pre-eclampsia, miscarriage, and delay in the intrauterine growth. For this reason, there are some authors that believe it is important to research Leiden factor V in women who have a family history of thromboembolism and wish to become pregnant.^6,7^

During pregnancy, the physiological state of hypercoagulability in association with the ATIII acquired deficiency increases the risk of pulmonary thromboembolism to 70%. Hence it is important to measure hypercoagulability parameters during gestation, such as ATIII and ATIII thrombin complex.^4^

Moderate or severe hyperhomocysteinemia is a risk factor for myocardial infarction, arterial disease or venous thrombosis in young people, and for recurrent venous thrombosis. Due to our patient's presentation of recurrent venous thrombosis, young age and increased homocysteinemia levels, it could be concluded that she had moderate hyperhomocysteinemia.

SLE was considered as a diagnostic hypothesis in this case because the patient was presenting nephrotic syndrome that started during the puerperium, in association with the presence of erythematous lesion on the face, Raynaud, reticular livedo, and arthralgia in the knee.^10^ Prednisone was introduced as a result of this diagnostic hypothesis, which was not confirmed because of the clinical evolution, the absence of clinical criteria, and the serological findings. Nevertheless, this diagnostic hypothesis may only be totally excluded after long-term attendance.

The antiphospholipid antibody syndrome (AFAS) is a clinical entity that leads to a state of hypercoagulability. It is characterized by recurrent venous or arterial thrombosis, fetal death through miscarriage, and thrombocytopenia associated with the presence of circulating antiphospholipid antibodies (AFA) represented by the anticardiolipin (ACL) and/or lupus anticoagulant (LA).^1,11^ In just 50% of AFAS cases are both the tests positive, which makes it necessary to perform the two tests in clinically suspicious cases. LA is considered positive when at least two of the following tests are positive: prolonged PTT that is not corrected with the addition of fresh plasma rich in coagulation factors;^14^ dRVVT (dilute Russell's Viper Venom test); and Kaolin coagulation clotting time. In this way, this antibody may be a risk marker for thrombosis or have an important direct role in the pathogenesis of this complication.

When validating the thrombus, the circulating AFA may be negative, which makes it obligatory to repetitively research the antibodies in the thrombotic phenomena.^12^ The relation between AFA and thrombosis was suggested by Bowie in 1963, and from then on, there have been many studies confirming this relation.^1,12^ It has been noticed that women with a history of recurrent miscarriages have 15% AFA incidence and a 90% probability of pregnancy failure.^1,12^ The complications in a gestation followed by the antiphospholipid antibody syndrome are arterial or venous thrombosis, thrombocytopenia, preeclampsia, toxemia, low fecundation rates, neurological, cardiological, renal and hepatic alterations, higher risk of postpartum depression and postpartum serositis syndrome.^1,12,13^ The renal complications associated with the presence of AFA are hypertension, renal insufficiency, renal venous thrombosis and renal infarction. Patients with positive AFA symptoms should receive anti-thrombotic therapy, although the time and length of the treatment are still not well determined. The recurrence risk for thrombosis is high after an interruption in therapy, justifying the prolonged anticoagulation. However, the hemorrhage risk cannot be neglected either. Occasionally, in some cases, the AF antibodies extend the PT and the INR, making proper control of the coagulation difficult.^11-13^

AFAS was considered in the case of this patient because of the presence of venous thrombosis and reticular livedo, and also because her sister presented a history of recurrent miscarriages and deep vein thrombosis. The fact that the patient had her parturition at full term, with absence of thrombocytopenia and anticardiolipin antibody, negative LA at first, and normal PTT, made this diagnosis seem improbable. However, as the patient presented recurrent thrombosis and positive lupus anticoagulant verified by many techniques, the diagnosis of antiphospholipid antibody syndrome was confirmed.

The gestational puerperal cycle is a transitory thrombotic state that may predispose to thromboembolic phenomena, whose major complication is the pulmonary thromboembolism, which may be fatal.^14^ Patients with thrombosis associated with a family history of this should be investigated (ATIII dosage, Leiden factor V, proteins C and S, homocysteine, AFA) and alerted to the risk of developing thromboembolic phenomena (Table) in the face of puerperium risk factors for nephrotic syndrome, surgeries, and immobilization.^14^

**Table:** Clinical states that may predispose to thromboembolic phenomena

Deficiency	Etiology	Clinical Condition	Diagnosis
ATIII Deficiency	Dominant autosomal heredity	Thrombosis started in the puberty or adult	Chromogen substrate
Protein C Deficiency	Acquired: nephrotic syndrome, anti-contraceptive intake, hepatic cirrhosis);	Venous thromboembolism	Protein C activity (immunoenzymatic or amidolysis of the chromogen substrate
Protein S Deficiency	Dominant autosomal heredity	Similar to the deficiency of protein C	Protein S free in the plasma
Leiden Factor V	Mutation in the point G1691A of the protein factor V		Activated protein C resistance; Molecular test
Hyperhomocysteinemia	Recessive autosomal heredity leading to an alteration of the enzyme cystothionin-b-synthetase Acquired: colabamine, folate or pyridoxine deficiency; chronic renal insufficiency, methotrexate or anti-convulsant intake	Mental deficiency, skeletal abnor-malities; arterial and venous throm-bosis; recurrent venous thrombosis; Risk factor for severe myocardial infarction	High pressure liquid chromatography
Antiphospholipid Antibody	Unknown	Antiphospholipid antibody syndrome (recurrent venous or arterial thrombosis; fetal death with miscar-riage and thrombocytopenia)	Positivity at least in these two tests: prolonged APTT, that is not corrected with the addition of fresh plasma rich in coagulation factors, dRVVT (dilute Russell's Viper Venom test) and Kaolin clotting time of coagulation)
